# Stories of Hope: Young People’s Personal Narratives About ADHD Put Into Context of Positive Aspects

**DOI:** 10.1177/10497323231206936

**Published:** 2023-11-02

**Authors:** Siv Vea Grønneberg, Eivind Engebretsen, Stine Torp Løkkeberg

**Affiliations:** 1 Faculty of Health, Welfare and Organisation, 3678 Østfold University College, Fredrikstad, Norway; 2 Institute of Health and Society, 6305 University of Oslo, Oslo, Norway

**Keywords:** attention-deficit/hyperactivity disorder, self-narratives, subjectivity, positive aspects, societal structures, norms

## Abstract

There is a growing acceptance that ADHD is a multi-dimensional disorder in which not all symptoms are associated with deficits or functional impairments. This article contributes to research on the positive aspects of the diagnosis, specifically understanding the positive aspects of living with ADHD. The empirical data was based on individual interviews and self-narratives of 10 young adults with ADHD. Narrative analysis was implemented when investigating their stories. The findings showed that challenges with the diagnosis were not necessarily stably occupied, and for some, the diagnosis was thought of as a benefit and something they would not have been without. Four stories highlighted particularly the context of positive aspects: (1) insight and strategies, (2) targeted efforts, (3) balanced energy, and (4) social skills. These aspects were correlated to both the individual’s strengths as well as the strengths and support that could be related to their societal and cultural environment.

## Introduction

People who live with attention-deficit/hyperactivity disorder (ADHD) “too often hear about the diagnosis in relation to deficits, functional impairments and associations with substance misuse, criminality or other disadvantages in almost every level of life” (Sedgwick et al., 2019, p. 250). The leading diagnostic manual in the Western world, the American Psychiatric Association’s *Diagnostic and Statistical Manual of Mental Disorders* (DSM), describes ADHD as a persistent pattern of inattention and/or hyperactivity–impulsivity interfering with functioning or development—symptoms that tend to develop in childhood and often persist into adulthood (American Psychiatric Association, 2013). Furthermore, the diagnosis is perceived as severely impacting quality of life as well as challenges with social and occupational functioning.

Opinions conveyed by reputable organizations, such as the American Psychiatric Association, have a significant influence over perceptions with ADHD due to their high position in the “hierarchy of credibility” (Becker, 1967, p. 242). This is related to the fact that narratives and discourses are influenced by political, religious, and cultural ideologies that produce “practices that systematically form the objects of which they speak” (Foucault, 1972, p. 49). In other words, such ideologies contain power relations that create social identities or “subject positions” for people and thereby are influencing how we understand ourselves and are understood by others. As such, the deficit focus in the leading manuals might affect the norm about who we should and ought to be–how we understand ourselves or are understood by others (Timimi, 2017). Erlandsson and Punzi (2017) emphasized that “The time has come to ask ourselves how the tendency to portrait children as dysfunctional has evolved.”

However, there is an increasing acceptance that several mental health conditions, such as ADHD, are seen as multi-dimensional disorders in which not all symptoms represent deficits or functional impairments (Epstein & Loren, 2013). A longitudinal study supported the dimensional aspect in highlighting that symptoms of ADHD existed along a continuum with fluctuations in symptom severity and developmental trajectories—and for some that the symptoms persisted into adulthood, while for others they attenuated (Biederman et al., 2012). The understanding that difficult situations can change and challenges can be overcome is central for resilience, optimism, future hopes, and well-being (Hertz, 2008; Rasmussen et al., 2009; Seligman & Csikszentmihalyi, 2000). Weiss (2016) emphasized that adults that meet the diagnostic criteria for ADHD can function quite well. Positive aspects attributed to high functioning (HF)-ADHD have been noted with some benefits, resources, skills, and strategies that can be used to mediate and/or compensate for ADHD-related deficits or impairments, such as hyper-focus and eidetic learning, by putting in twice as much effort in than others (Lesch, 2018). Mahdi et al. (2017) described positive aspects such as energy and drive, creativity, hyper-focus, empathy, agreeableness, and willingness to assist others. They concluded that there was a need for further clarity and understanding beyond the diagnostic criteria.

In the search for literature about positive aspects and treatment practices related to ADHD, the articles found mainly focused on the standard treatment approaches of ADHD rather than any empirical research addressing the positive aspects or approaches to support empowerment and personal strengthening (Lesch, 2018; Seligman, 2011; Timimi, 2017). In this regard, Lesch (2018, p. 191) emphasized that it is time “to intensify discussions about how research on ADHD can be shifted from the deficit-focused view to a concept that is orientated towards resources a patient might be able to utilize.” Focus on positive aspects also provide support for overcoming challenging situations which are crucial for future hopes and in “predicting a broad range of well-being outcomes including psychological well-being, social well-being, and subjective well-being” (Murphy, 2023, p. 3).

The research content within this article used narrative analysis to focus on understanding the individual’s own stories when asking: What are the stories of the positive aspects of living with ADHD about? What are the orientations, characteristics, and actions behind these stories? What possibilities in life do the stories reflect? What norms and recourses are reflected in the individual’s cultural and societal environment?

By including the voices of those that the stories are about, those who represent a group or category that can be understood as marginalized, one can establish a new basis for power that might challenge the existing view in the hierarchy of credibility (Becker, 1967) and, therefore, change prevailing norms and taken-for-granted perceptions in society. The aim was to promote beliefs of personal engagement and developmental efforts related to enabling and to have optimistic hopes for the future.

## Method

The approach used in this study was inspired by principles of classic narrative analysis and discourse analysis. When aiming to explore individuals’ self-narratives, narrative analysis became useful as it, by its nature, focuses explicitly on the study of narratives told during interviews (Brinkmann & Kvale, 2015). Gergen (2001) explained self-narrative as personal stories that span in time and that depict one’s identity and the causes behind a current condition. How self-narratives are organized provides different opportunities for action and opportunity domains in the present and in the future, which can be both limiting as well as evolving (Jansen, 2013).

To identify the narratives that could be related to positive aspects of living with ADHD, the analysis is inspired of Labov’s six-step analysis (the abstract, the orientation, the complicated action, the evaluation, the resolution, and the coda) (Johansson, 2005). In this study, we choose to use the following steps: (1) the abstract (what the story is about), (2) the orientation and action (characteristics and conditions), and (3) the result and evaluation (the outcome and possibilities in life). At step (4), termed as “relation to societal structures and norms”, we draw on discourse theory/analysis. These steps, and the combination of approaches, will be elaborated more in the Data Analysis section.

While narrative theory focuses mainly on the person and his/her personal accounts, discourse theory is more concerned with subject positions and the relationship to norms and societal structures inherent in language (Snow, 2001; Winther Jørgensen & Phillips, 1999). Boréus and Bergström (2012) underscored the importance of exploring narratives as a mean to enhance our comprehension of culture and society. They argued that examining narratives within the context of an individual’s cultural and societal surrounding can yield valuable insights. This exploration was facilitated by incorporating elements from discourse theory. Chatman (1975) further bolstered this methodology, emphasizing that a narrative inherently includes a discourse, reinforcing the relevance of integrating these two approaches. A concept within discourse theory is that one truth might exclude another and that new ways of thinking can emerge by finding what a certain discourse excludes (Foucault, 1971). In this regard, to be aware of exclusion mechanism in discourses is also to be aware of what is not said (Winther Jørgensen & Phillips, 1999).

Foucault (1971) claims that subjects are created from discourses by identifying with specific ways of being and distancing oneself from others. In the context of discourse theory, those understandings that win with their truths also contribute to shared “we-ness” in “the hierarchy of credibility” (Snow, 2001; Winther Jørgensen & Phillips, 1999). In other words, such identifying might lead to interactive senses of “we-ness”, which can be understood as collective identities related to certain norms and societal structures that are interpreted as the truth (Snow, 2001). This perspective provided an opportunity to focus on the “contextual opportunities and restrictions of how a person negotiates who to be within certain discursive terrains” (Jansen & Andenæs, 2013, p. 122).

### Selection

Ten potential participants were sent an invitation letter asking to share their experiences of living with ADHD. This was after information about the project was sent to three high school counsellors and three counsellors at a college. These were counsellors that through their work and network had contact with young people with ADHD and the ones that established the first contact with relevant candidates. Those who responded positively about participating received the invitation letter describing the project, the informed consent procedures, and an information sheet that had to be signed in order to participate in the study. Also, two participants were recruited through the “snowball method” as they were suggested from two already recruited participants. The selection process occurred continuously and in parallel with the ongoing data collection, until we concluded that the material had sufficient informational strength to answer the research questions. Those who contributed to the recruitment were then informed that the project had enough participants.

The selection was strategic in that there were guidelines in relation to age and to equal distribution of gender. The main criteria for inclusion were to have received an ADHD diagnosis. There were no specified exclusion criteria, for example, there were no intention to exclude informants with “double diagnoses.” However, the participants were selected from high schools and colleges and in that sense had experienced a particular “success” with regard to schooling.

The overarching topic for the inquiry was “How am I me?” and aimed at the participants experiences of living with the diagnosis in general. Findings of narratives that could be related to negative characteristics and actions were dominating. However, experiences that initially had a negative connotation also turned out with information and wisdom for later agency and coping. Findings of positive aspects were also to be found in what was in the implicit and implied of statements (which will be explained more in the Data Analysis section). As such, this study’s interest for the experiences about the positive aspects came up as something that grew organically from the transcriptions and which inspired this article.

The participants, five young women and five young men, were in an age range of 18–28 when the interviews were conducted and in the range of 6–16 years old when they were diagnosed. Most were diagnosed based on concerns from parents or schoolteachers about concentration difficulties and/or unwanted behavior. However, two of the participants initiated the request to be assessed for the diagnosis in their teens.

They were demographically similar as they all came from the same economic class, the same part of Norway, and had the same ethnic background. The average age when the interviews took place was 22, the average age when diagnosed was 10, and it was on average 10 years since they were diagnosed. They had all completed upper secondary school, vocational education, or were students (one had completed an artistic bachelor’s degree). All were medicated when they received the diagnosis. At the time of the interview, five were still receiving medication on a daily basis, three used medications occasionally (such as in relation with exams), and two were not using medication at all. None received treatment beyond medication when they received the diagnosis. However, when the interviews took place, all the females and one of the males had either applied for, attended to, or continued to attend therapeutic counselling with a professional therapist, and also cognitive therapy was noted.

One researcher conducted the interviews in suitable places near the individual’s location or in the individual’s home, and three of the interviews were conducted digitally through Zoom. The individual interviews were semi-structured, with room for leaps of thought and digressions, and had the form of a conversation. They lasted from 35 to 70 minutes, with an average time of 55 minutes, and were audio recorded and transcribed directly after completion.

### Ethics

All names in this study are fully anonymized, and other details which could lead to the identification of a participant have been changed or omitted. Informed consent was obtained from all individuals that participated in this study before the interviews commenced. The Regional Committees approved the study for Medical and Health Research Ethics (REK) (ref: 2019/85) and Norwegian Centre for Research Data (NSD) (ref: 216926).

### Data Analysis

NVivo 12, a program for qualitative research, was used as a tool for the analysis. The audio files were transcribed in the program, and a case was created for each of the participants.

**Figure 1.**
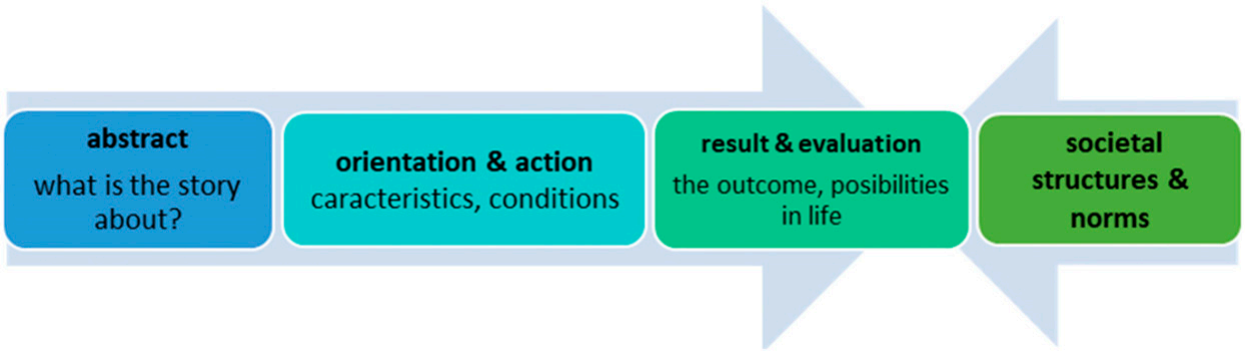
An overview of the steps in the analysis and how the combination of the two approaches is used to explore the content and outcome of the stories told.

The analysis was carried out in four steps. The first three steps, the “abstract”, the “orientation and action”, and the “result and evaluation”, were inspired by steps in narrative analysis (Labov & Waletzky, 1997). The last step, “societal structures and norms”, was drawn from discourse analysis (Foucault, 1971). Figure 1 presents the four steps in the analysis and how the two approaches are used to explore the content of the abstracts and the outcome of the stories told.

The analysis started with seeking an overview of what the stories were about—the abstract—“a recapitulation of past experience that match a verbal sequences of clauses to the sequence of events that actually occurred” (Labov & Waletzky, 1997, p. 12). The transcripts were read and reread several times to identify narratives that dealt with coping resources. These were marked and drawn into a folder in NVivo 12, which ended up constituting 23 sub-expressions. After reviewing the sub-expressions, we found that the content could be gathered into four key abstracts that made up four corresponding folders in the program. These were (1) Insight and strategies for managing one’s situation, (2) Targeted efforts and educational goals, (3) Balanced energy, and (4) Social skills. Relevant quotes from the transcriptions were marked and drawn into the corresponding folder that followed each abstract. The program kept track of which person the quotes belonged to, which made each participant “visible” for the analysis with their unique experiences.

Secondly, we searched for the “orientation and action” within these abstracts. This step provided information about the initial events, characteristic, or/and condition—“what happened before the event occurred.” Furthermore, the “action-part” refers to the point where certain events, characteristics, and actions move the story ahead (Labov & Waletzky, 1997).

Step 3 in the analysis, the “result and evaluation”, appears as a merger of Labov’s third and fourth step (evaluation and resolution) (Johansson, 2005). This step refers to the result or the outcome—to the meaning of what has been or could have been helpful and what challenges that might lay behind achievements and, furthermore, to the individuals experiences of possibilities in life, both in the present and in the future (Jansen, 2013).

At step 4, we searched for relations between the outcome of the abstracts and the individual’s societal and cultural environment. For example, receiving help from mother also pointed to the mother as a central resource in the individual’s environment. When we searched for collective identities, or so-called shared “we-ness”, we focused on Foucault (1971) who claimed that subjects were created through discourses by identifying with specific ways of being and distancing ourselves from others.” For example, when one of the participants says “(…) I usually say that we’re lucky to have it (ADHD) (…)”, this also indicated a role one identified with.

Throughout the analysis, we focused on statements that referred to narratives that were articulated directly, for example, when participants referred to an energy by saying “I like my energy because I get to accomplish a lot during the day.” According to discourse theory, we also searched for what was implicit and implied in the statement (Boréus & Bergström, 2017). For example, when a participant says that his energy makes him feel like “superman”, this also refers to an experience of strength in, for example, being “someone who gets things done.” We also focused on the discourse analysis principles of exclusion mechanism (Foucault, 1971). In this regard, a saying with a negative intonation such as “the only reason I managed school was because I received a lot of help from my mother” could exclude some “truths”—truths that in the implied could point to strengths in also being one who actually made it through school and one who was willing to do the effort needed.

## Results

Although the participants in this study most often referred to narratives about difficulties, these narratives were not necessarily completely negative or stably occupied. There were also positive aspects regarding characteristics and actions to be found in what was implicit and implied in the narratives told and as something that appeared hidden for the individual. Some experienced their diagnosis as a resource they would not be without.

### Step 1: The Abstracts—What Were the Stories of Positive Aspects About?

Through narrative analysis, we identified four key abstracts, which contained 23 sub-expressions, which were related to experiences of living with ADHD that reflected specific coping resources and characteristics that gave agency to handle their own lives. These were (1) insight and strategies, (2) targeted efforts, (3) balanced energy, and (4) social skills (Table 1).

**Table 1.** The Four Key Abstracts and Related Sub-Expressions.

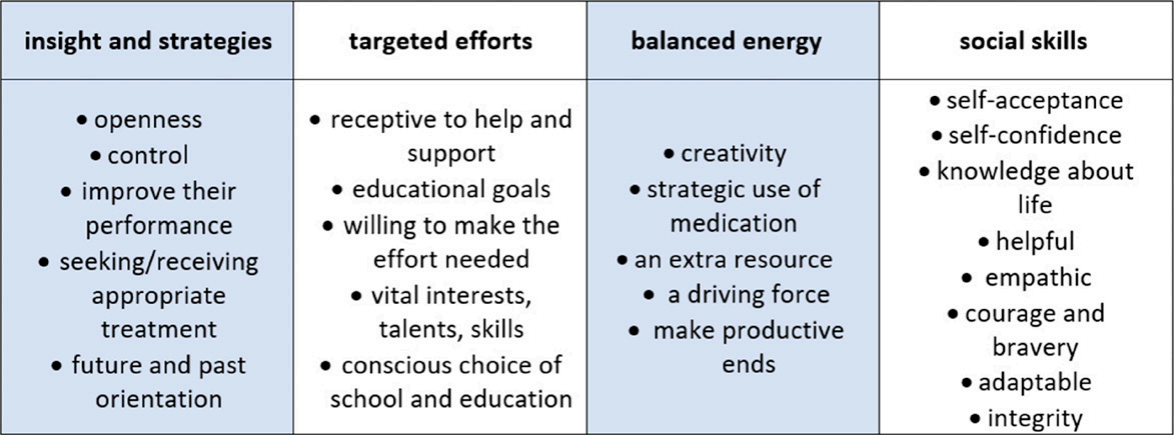

The content under each abstract cannot be regarded as an absolute as there were variations, for example, openness, courage, and being adaptable that were reflected in several of the abstracts. The content of the abstracts will be presented more elaborately in the following.

### Step 2: The Orientation, Characteristics, and Actions Behind the Abstracts

#### Abstract 1: Insight and Strategies to Handle Own Situations

Several had experienced lack of coherence and control as well as frustrations, especially during childhood and adolescent years, that led them to search for insights and tools to handle their situations. All experienced being medicated when they received their diagnosis. One received shortly after diagnosed talks with a professional therapist, which was sought privately. Most said that medication helped them with concentrating and better control but not with insight and tools to handle their situations. Nina, one of the participants, stated that she in retrospect thought her family needed support—that her home condition should have been more closely looked at when she received the diagnosis. She believes that such support would have helped her feel better at an earlier stage in life: “I have had an unstable home with a sick mother … and conflicts … and, in a way, unsafe in that sense. If these conditions had been looked more into, the situation might have been different (…).”

At the time of the interview, one of the young men and all the young women had either applied for, attended, or continued to attend therapeutic counselling with a professional therapist. Two participants, attending bachelor programs at two different colleges in Great Britain, were offered cognitive behavioral therapy as part of a program at their respective institutions. Hilde stated that she was put straight on medication when she received the diagnosis and said that she wished she had attended behavioral therapy much earlier in her treatment, as it helped her positively with “ways to think, and of learned helplessness and how to act on habits and routines.”

Mikkel is a successful trainee who was headhunted for a steady job in a well-reputed company. He stated that he only used medication, when necessary, primarily when he had to take exams. He had developed routines and strategies to avoid distractions and to concentrate on all tasks required at work:

When reading for exams … there were a thousand other things to do, such as putting on a machine with laundry, cleaning the room. Now, I drive up to the office where there is nothing to distract me. I’ve become more aware like that. (…) I get daily many messages from my supervisor, and things are changing fast … so, every day I sit with this book (lift up a book) and I write up everything which I consecutively iron out when it's done.

The rigid self-organization also appeared as a tool for maintaining order and allowed for greater achievement with daily routines. However, these rigid organizational structures were for some prone to setbacks. For Ylva, the establishment of routines appeared both necessary and rigid. She says, “I have fixed routines about where I put things. If not, I’m running around and being completely hysterical.”

#### Abstract 2: Targeted Efforts

All the participants responded that they through their upbringings had experienced a certain pressure as being the ones responsible for adapting to their surroundings rather than the other way around. Erlend, who worked in a store, said he was tired of the “educational race” and thus decided to forego further studies. He stated that he believed he would have been better off a hundred years ago when there was less academic pressure.

The orientation toward this abstract was initiated mainly through the help they had received from both formal networks (e.g., at school and counselling) and informal networks (e.g., family and friends), which had both been crucial in, for example, making it through school and getting a driver’s license. Those who highlighted that they had received support and recognition for their efforts at school and/or home tended to be those who found an educational way to use their interests, talents, and skills.

Per emphasized that the help he got from an assistant teacher was crucial for him in going back to school after dropping out for some time: “I was offered complimentary breakfast every morning, and I had a supervisor at school who called me every day and messaged me asking how I was doing. He made a fantastic effort and helped me move forward after I dropped out.”

Mikkel and Per acknowledged that they would not have made it through the school years without the help they had received from their mothers. Per was now reading for his driving test and felt that he now would manage to pass because his girlfriend ensured that he practiced: “Luckily, I’ve got a girlfriend who’s super strict and tells me that now you’re going to practice, so now I must sit and practice on that app on the phone, and it’s gone well … so now I think I can make it happen.” Despite negative intonations when referring to themselves in these sayings, the implied in these statements also referred to specific strengths, such as being one who is adaptive to help, one who can appreciate help, and one who (despite challenges) is willing to make the effort needed as well as one who made it through school. “Hidden” strengths in the example above also reflect good hope in being one with an opportunity to succeed in passing the driving test.

The motivation behind such effort varied at different times. Nina stated that she today loves her study subjects and career choice despite having to spend twice the amount of time on schoolwork compared to her classmates. She initially made such an effort because she was afraid of appearing different from “the others” due to being academically behind “the others.” Mikkel stated he was held on tight reins by his mother in elementary and middle school. However, today he has gained certain strategies such as making a daily list of “what to do.” He is very motivated in his trainee position, where he puts in much effort: “I never end the workday regardless of whether it’s five or eleven o’clock in the evening until I’ve ironed out everything (to be done) in the book.”

The findings indicated a willingness and ability to put in as much effort as needed, even more than “others”, to reach their targeted goals. In addition, the choice of school and pedagogy appears to have significantly influenced mastery and recognition. For example, Cecilia emphasized the fact that she had attended a Waldorf school^1^ and later chose a bachelor which was practically and artistically orientated made her path of attaining her educational goals toward an artistic profession easier.

#### Abstract 3: Balanced Energy

Several referred to a time when they had experienced their energies as being “out of control”, and some mentioned that they missed this energy level. For some, the power had taken a “path” that had made it become positively attributed to their diagnosis and claimed it as an extra resource in life.

Nina was studying to become a social worker while at the same time, she was running her own company that sold products on internet. She stated that she felt lucky to have the diagnosis, which she related to as her energy: “It is a driving force that helps me make things happen, to do what I want, and to follow my dreams (…). I usually say … that we are lucky to have it (ADHD). I see it as a strength, for it has helped me to get where I am today (…).”

Mikkel is a trainee in a successful company and referred to his energy as an advantage for his achievements: “I feel … kind of like Superman, I always have an extra gear, so when it’s 4 o’clock and the office is empty, then I’m happy to sit for a couple more hours, I see it as an advantage.” This “cornucopia” of energy was perceived as a resource for achieving and doing more than others; Mikkel further stated, “I live with a buddy who has the same job as me. When he is home at 7 p.m., he is exhausted and lying flat on the couch. I am not tired even if I come home 2 hours later—I am ready for town.”

Energy considered an extra resource was particularly highlighted for those who found that it supported their behavior, was creative, and occupied their attention with intense solid interest. Those who reported such energy were also those that used medication (for ADHD) only when needed, in situations that demanded extra concentration, for example, about exams and daily lectures. Nina stated that such a regulation was necessary because she lost energy and says she became “more A4”^2^ on medication. Not wanting to be A4, or “ordinary”, refers to courage and bravery in not being afraid of standing out or being different from “the others”, which in the implicit also reports both self-confidence and integrity.

#### Abstract 4: Empathy and Social Skills

Most had experienced periods of social insecurity, which also reflected experiences of shame and guilt. Some made a great effort not to stand out from others by remaining behind with school subjects. Several had experienced lots of arguments with parents and siblings, insecurity about classmates, and roles expressed as, for example, “I was the class clown”, “the scapegoat”, and “an outcast.” Experiences of such roles made it essential for Per to start with “clean slates” after completion of the primary and secondary schools where such positions were most evident, which implicit reflected both bravery and a “can-do” attitude—and an orientation away from roles that felt inhibiting.

The findings revealed transformations in narratives where social and emphatic skills were emphasized, from being, for example, the “scapegoat” to one with knowledge of life or from “being one who stands out from others and being weird” to “one who is confident and has resources that are helpful to others.” For example, Per, who worked with children and adolescents with behavioral-related diagnoses, stated that his experiences with the diagnosis had become a resource he utilized daily: “I can convey confidence to the children around me. It’s always a good feeling when someone who’s often angry, frustrated, and tired comes and leans their head against your shoulder; then you know you’ve done something right.” Ylva said she was no longer so concerned about what others thought and that she had gained experiences that were helpful to others*:* “(…) I want to help others to feel as good as I do now (…). I have a friend, who sent me a message asking about my experience, and if I had any advice that I could give her. Then, I felt I was useful to her.”

### Step 3: Result and Evaluation—The Outcome and Possibilities in Life

The four abstracts above also reflected an outcome in referring to strategies that worked, insight and knowledge of life, targeted efforts, balanced energy, and social skills. The content in the abstracts pointed further to certain achievements and possibilities. Key elements in this regard were (a) control and improved performance, (b) self-confidence and integrity, (c) accomplishment of goals, and (d) that one can achieve the same or more than “others.”

Achievements of control and improved performance reflected the capacity for strategic thinking, which was particularly evident in being able to create routines and habits that worked, especially those that also persisted when they were without medication. The ongoing search for strategies and insight indicated curiosity and openness which pointed to both, an orientation to the past and toward the future. That one learned from “mistakes” and could improve performance in current problematic areas as well as in the future pointed to the ability to adapt to one’s surroundings as well as to positive hopes for the future.

Self-confidence and integrity, as well as bravery and courage in not being afraid of being different from “the others”, were highlighted through statements, such as when Ylva says that she was no longer concerned about what others think of her, and Nina says she liked herself in not being “an ordinary person.” Being the one that other sought advise from was highlighted as something that gave them experiences of acquired competence in life—a social capital that were useful for other young persons in similar situations. Such achievement reflected social skills and empathy, as well as mastery and sense of meaning in life.

Those who experienced energy as an extra resource also reported creativeness and solid interest. Creativity and free flowing of ideas pointed to non-formal thinking, the ability to think outside “the box”, which was particularly evident for Cecilia in her artistic profession. To be noted is that those who controlled their medication by taking it on special occasions were the same as those who reported that they could turn their energy into a meaningful direction and something productive. Experiences of coping and intrinsic motivation appeared as a force that maintained a positive sense of self, which made it possible to achieve one’s goals—that one could achieve the same or more than others.

### Step 4: Relations to Societal Structures and Norms

The abstracts and the outcome in the steps above also reflected influences of specific societal structures and norms in the individual’s cultural environment. Those who received recognition and support at school and help from their mothers or girlfriends to remediate functional impairment reported increased self-care skills that helped them prepare for “the future.” In this regard, offers of conversations with a professional therapist and psychoeducation were highlighted, as well as self-regulated use of medication.

Individual adapted pedagogy and requirements at school and a career choice where they got recognized for their interests and talents were highlighted as efforts that contributed to achieving their educational goals—especially for those who were practically and artistically oriented. These findings reflected the importance of a supportive environment and arenas facilitating interests and talents. Most statements related to social well-being had self-acceptance and self-confidence as positive attributes and were related to experiences of being accepted, which in turn pointed to the importance of an inclusive environment.

The findings of shared “we-ness” indicated influences of certain societal norms about ADHD that represented shared belief systems. For example, when Nina used the term “we”, saying, “I usually say that we’re lucky to have it (ADHD)”, she reassured herself, in a (positive) way that indicated that she identified with a collective history and associated herself with some prevailing perceptions of a social group, expressly “we who have ADHD.” When Mikkel, on the other hand, stated, “I was never the typical one (with ADHD), the one who sat on the last bench, made noise and overturned the desk”, he was suggesting there was a collective history with having the diagnosis, which he identified as something that had a prevailing perception and as a result a norm he distanced himself from. This finding referred to the narratives and abstracts of agency. It depicted the individual’s ability to put their narratives into stories that reflected value for one’s well-being, which also should be added to the actions and characteristics behind the positive aspects.

Table 2 presents an overview of the characteristics found in the individual’s societal and cultural environment regarding achievements and possibilities in life that apparently had an impact on future hopes.

**Table 2.** Characteristics in the Individual’s Societal and Cultural Environment That Had an Impact on the Key Abstracts and Outcome.

Characteristics in the individual’s cultural environment			
• Acceptance and support at school		• Family support	
• Home advantages		• Adapted medication	
• Adaptable requirements		• An inclusive environment	
• Adapted pedagogy		• Arenas for interests and talents	
• Occupation for interest and talents		• Artistic and practical subjects at school	
• Therapy/psychoeducation		• Norms one can relate to and distance from	
Insight and strategies	Targeted effort	Balanced energy	Social skills
The possibility to achieve the same or more than others			

## Discussion

The purpose of this article has been to gain better insight into stories of young adults with ADHD that can be associated with positive aspects of the diagnosis and that can reinforce strengths and skills that enable coping and well-being.

The findings highlighted that most plots, actions, and conditions behind the actual abstracts and possibility domains initially had a negative connotation. However, the “failure” experiences contained information and wisdom for later agency and coping, even when initial success was hard to spot. These findings emphasized that their challenges and limited positions could be handled and overcome, which could significantly influenced future hopes (Murphy, 2023; White, 2007).

There was also an account related to narratives that indicated strengths that were to be found implied and implicit in the statements (Boréus & Bergström, 2017). For example, when a narrative that initially appeared stated as unfavorable, like “the only reason I managed school was that I got a lot of help from my mother”, and the same narrative also revealed strengths in being “one who was receptive to help”, “one who was able to put in the effort needed to accomplish the tasks”, and also “one who made it through school.” Theory regarding narrative practice, a common approach utilized with family therapy, emphasizes the advantage of lifting the implied to create parallel stories of strengths—reflections that could help to turn a “problem story” or a “thin” story into a “thicker” story—a story that focuses on resources and what works (White, 2007). In this regard, findings pointed to the need for the individual’s supporters and the individual to be aware of such strengths because what is being implied may be hidden from the individual (White & Epston, 1990). In sum, the four key abstracts reflected certain individual strengths and strengths that could be related to the individual’s cultural environment.

### Individual Strengths

Findings of positive aspects reflected curiosity and openness to experiences toward learning and to receive support that gave rise to insight and strategies. These characteristics allow one to pursue ambitions and discover meaning in one’s own life (Zuss, 2012), which have an essential role in influencing well-being (Sheldon et al., 2015). Furthermore, the ability to learn from one’s mistakes has led to experiences of better self-regulation and control which are characteristics that contribute to resilience and protective forces that support well-being (Murray, 2015).

Findings highlighted that strict routines reinforced feelings of control in one’s daily life as well as feelings of confusion and stress when these routines failed. Lesch (2018) highlighted that individuals with HF-ADHD would put much effort into control and often felt humiliated by setbacks. Therefore, setbacks should be known as an inevitable part of any recovery process and not as a state of giving up (Timimi, 2017), and instead be seen as something natural that can promote development, depending on how such stressors are dealt with and handled (Gassne, 2008). Struggles that appear manageable contribute to resilience and integrity in taking responsibility for one’s own life (Sedgwick et al., 2019), factors which are central for developing a positive self-image (Fleischmann & Miller, 2013).

Findings referred to social skills that reflected bravery and self-acceptance in, for example, not being afraid to stand out from others, which also pointed to integrity (Carter, 1996). Such resistance to other’s opinions of who they were appeared as both self-protective and self-enhancing. Thoits (2011) highlighted that some individuals are more resistant to mental illness stigmatization and stereotyping than others, as they reject others’ damaging remarks and behaviors and refuse to see themselves as others perceive them.

The continuous search for insight made them more personally reflective and sensitive to recognize emotions within themselves as well as the emotional states of others, which appeared as a protective factor for well-being (Sedgwick et al., 2019). Being helpful and emphatic to others was stated as something that made the participants feel useful and gave mastery and a sense of meaning. These findings aligned with the studies that reported positive aspects related to ADHD, such as agreeableness, empathy, and willingness to assist others (Mahdi et al., 2017).

Intrinsic motivation and energy were particularly highlighted in those who were creative and occupied or focused on interest, and as something that gave them the ability to both manage and achieve more than others. These findings agreed with the studies that emphasized that intrinsic motivation and mental spirit are resources in achieving one’s goals in life (Sedgwick et al., 2019; Von Culin et al., 2014). In this regard, Piumatti (2018) emphasized that intrinsic motivation serves to protect against poor mental health. Another advantage of curiosity and vital interests is that this aligns with experiences of being in a flow state that contributes to one’s subjective well-being (Csikszentmihalyi, 1997; Tan et al., 2021). Those who reported their energy as an additional resource tended to be those who managed to put their energy into productive ends and those who controlled their energy by taking their medication for ADHD only when needed, mostly during school examinations. How they acquired this skill should be researched further because harnessing one’s energy for productive ends is desired in adults with ADHD (Sedgwick et al., 2019).

Grue (2013) argued that being identified with a disability came from external pressures that closed and opened specific opportunities. For example, when Nina referred to her energy and stated, “(…) I usually say that we’re lucky to have it” or when Per stated, “At school, I was never the one at the back row that turned the desks”, at the back row in the classroom, they also refer to a “we-ness”, or to some collective identities, regarding the diagnosis, certain norms, and expectations that they both could relate to or distance themselves from. The utilization of narratives strategically supported that identity could be built from within and ascertained when it was in one’s best interest. In this way, the findings aligned with Grue (2013) when suggesting that the narratives and abstracts of agency allowed the individual to put their narratives into useful stories for their well-being. This could also be related to social identity theory, where the individuals define their own identities with regard to social groups, and this could benefit and bolster self-identity (Islam, 2014). For instance, the participants could define their own identities with regard to one’s in-group (i.e., ADHD) and that they have the tendency to view one’s own group with a positive bias compared to the out-group. This could help the individuals to identify with a collective and depersonalized identity based on being a part of others with ADHD that is imbued with positive aspects (Islam, 2014).

### Strengths Related to the Individuals’ Cultural Environment

Support from formal and informal networks, for example, from a teacher who saw you, and/or especially having a “home advantage” with backing from mother and girlfriends, were highlighted resources for managing and accomplishing tasks, academic mastery, and a resource that supported targeted efforts. Support that enables one to find one’s niche is found to be central to the transition from risk to resilience for HF-ADHD in adulthood (Lesch, 2018). The “home advantages” also indicated that their families or “important others” had adapted to their situations in ways that facilitated well-being (Williamson et al., 2009).

None of the participants were offered treatment beyond pharmacological treatment when they received the diagnosis. At the time of the interview, all the young women and only one of the young men had attended or had applied for additional treatment. The male participants especially highlighted help from their mothers and girlfriends, which could be protective factors. On the other hand, these findings align with international studies that emphasized that young men experience barriers when seeking help for their struggle (Lynch et al., 2018).

Findings highlighted therapy that supported the establishment of positive, insightful ways of thinking was experienced as especially useful when performing tasks and avoiding clashes with others. In this regard, the findings supported a systematic review of pharmacological and non-pharmacological treatments, suggesting that adjunctive therapy to medication, for example, behavioral therapy, should be emphasized and adjusted to the need as well as stimulants (Catalá-López et al., 2017). Findings aligned with the study that pointed to the need for family support, since families experienced consistently that they were left on their own after the child was diagnosed (Olsvold, 2014).

The choice of pedagogy, the opportunity for practical and creative issues, and the selection of a career where they could utilize their talents appeared strongly related to their well-being. Hence, findings pointed to the importance of an educational system that facilitated creativity, artistic and practical subjects, and extracurricular activities that enabled, for example, sports and artistic activities, and added to the participant’s positive sense of well-being (Long et al., 2015). Creative activities, for example, drawing, were noted to increase attentional abilities and decrease impulsive behaviors over time, to enable better decision-making, better completion of tasks, and general growth at a developmental level (Smitheman-Brown & Church, 1996). Also, divergent thinking—the ability to generate creative ideas—is an “important ingredient for future episodic thought” (Thakral et al., 2021, p. 17). In this regard, findings pointed to the contradiction in the widespread minimization in the content of practical and creative subjects in primary schools in Norway since the 70s (Djupedal, 2022).

To address support for the specific challenges and strengths faced by each person with ADHD, finding supported Sonuga-Barke et al. (2023, p. 523) in emphasizing the importance of reflecting on the underlying multiform and dimensional structure of ADHD in relation to “the role of normative environmental-mental constraints in determining associated functional impairment.”

### Study Limitations and Strengths

Analyzing narratives and discourses can be complex, and there may be multiple valid interpretations. As with any study, our theoretical framework can be a limitation, as it might highlight some aspects of the material and obscure other parts.

A fundamental limitation is that one’s own biases, experiences, and preconceived notions can influence the researcher. Morse (2015) highlighted the potential influence of a researcher subjectivity when it came to the interpretation of data. Such subjectivity might affect both the meaning extracted from the text and the interpreting of the result (Boréus & Bergström, 2012).

To minimize such impact, careful thought was made in following methods and procedures that are typically used in qualitative research. Supervisors continuously evaluated data collection, materials, methods, findings, and results. Prior to this study, to ensure that the research questions were understandable and feasible, the questions were evaluated by young people with ADHD (male and female and in the same age range as the participants in this study).

The participant’s experiences appeared to be similar perhaps due to the fact they were mainly from the same part of the country, had the same ethnic background, and much of the same economic class. As such, the findings may not fully capture the full complexity and nuances of the research topic, leading to potential oversimplification of findings (Morse, 2015). A sample of 10 interviews may be considered relatively small and could limit the generalizability of the findings to a larger population. Another issue is that since there are on average 10 years since they were diagnosed, there may be recall bias due to time and memory failure.

The participants were not recruited to highlight positive aspects of living with ADHD; however, they were invited to contribute with their experiences of living with the diagnosis in general with the overarching topic “How am I me?”. Another issue was when searching for stories of personal strength, we searched also for stories that were implied or implicitly stated. These may be “truths” that are “hidden” from the individual—of which the individual is not aware of and therefor does not recognize that the experience possessed such strength. In this regard, we argue that to identify and to be aware of such implied narratives of strengths are crucial, as they contain hope and provide an opportunity for making such stories “richer”—to change prevailing narratives that are defined by problems (White & Epston, 1990).

A strength of the study’s design was that it favored the individual’s perspectives and versions of events, arguing that the positive aspects found might have relevance to other young adults with ADHD regardless of sample size, age, gender, or ethnicity. Furthermore, the findings aligned with former publications that reported positive aspects of ADHD (Lesch, 2018; Mahdi et al., 2017; Sedgwick et al., 2019). This did strengthen the reliability and the transferability of the findings.

### Summary and Conclusion

The findings showed that the females had sought counselling for their challenges and reflected more on the importance of understanding one’s surroundings and recognizing the *early* symptomatic stages. In this regard, the findings called for more awareness and support for boys and young men as they experienced the same challenges. Also, skills in harnessing one’s energy for productive ends should be considered for further research. Furthermore, these findings highlighted the need for more focus on practical and creative subjects’ availability in the educational system to prevent the concept of academic failure.

Self-narratives also reflected certain societal norms and expectations that pointed to collective identities related to the norm of ADHD—norms that the participants both related to and distanced themselves from. Individual strengths were reflected in the ability to put their narratives into stories that were deemed “good” and interpreted as applicable for their own well-being. Furthermore, findings suggested more awareness of the impact of norms and societal structures when it comes to support strengths, as well as to determine associated impairment related to ADHD.

The findings could be compared between a “top-down-power”, which referred to societal norms and structural systems (which govern on how one is understood and understands oneself), to a “bottom-up-power”, which represented the voice of those living with the diagnosis. However, these two types of power are not mutually exclusive. The second type of power may be the best option to guide the first when supporting the ability to create positive possibility domains and hopes for the future.

This article reaches out young people with ADHD to family members, teachers, practitioners, health services, and politicians to better support “good” narratives that work. Furthermore, we hope this knowledge can inspire and highlight ways of understanding oneself and one’s own life, which in turn provides the opportunity to focus on strengths and replace stories that do not work as well for mental well-being.

## Notes

1. The Waldorf approach toward learning draws on principles based on a pedagogical anthropology initially developed by Rudolf Steiner, and the idea of learning is based on the development of the person and guided by an ethics of care (Rawson M., 2022). L2 teaching and learning in Waldorf schools—why performative? *Scenario (Cork)*, *16*(1), 1–21. https://doi.org/10.33178/scenario.16.1.1.

2. A4 refers a standard paper size and is a Norwegian term for being “ordinary.”
